# Genome-Wide Analysis Reveals Key Genes and MicroRNAs Related to Pathogenic Mechanism in *Wuchereria bancrofti*

**DOI:** 10.3390/pathogens13121088

**Published:** 2024-12-10

**Authors:** Caoli Zhu, Yicheng Yan, Yaning Feng, Jiawei Sun, Mingdao Mu, Zhiyuan Yang

**Affiliations:** 1 School of Artificial Intelligence, Hangzhou Dianzi University, Hangzhou 310018, China; 2 School of Medical Technology and Information Engineering, Zhejiang Chinese Medical University, Hangzhou 310053, China; 3 School of Medicine, Southeast University, Nanjing 210009, China

**Keywords:** *Wuchereria bancrofti*, pathogenic mechanism, microRNA, genome-wide analysis

## Abstract

*Wuchereria bancrofti* is a parasite transmitted by mosquitoes and can cause a neglected tropical disease called Lymphatic filariasis. However, the genome of *W. bancrofti* was not well studied, making novel drug development difficult. This study aims to identify microRNA, annotate protein function, and explore the pathogenic mechanism of *W. bancrofti* by genome-wide analysis. Novel miRNAs were identified by analysis of expressed sequence tags (ESTs) from this parasite. Protein homology was obtained by a bidirectional best-hit strategy using BLAST. By an EST-based method, we identified 20 novel miRNAs in the genome. The AU content of these miRNAs ranged from 39.7% to 80.0%, with a mean of 52.9%. Among them, 14 miRNA homologs were present in mammal genomes, while six miRNA homologs were present in non-mammal genomes. By conducting a detailed sequence alignment using BLAST, we have successfully annotated the functions of 75 previously unannotated proteins, enhancing our understanding of the proteome and potentially revealing new targets for therapy. Homology distribution analysis indicated that a set of critical proteins were present in parasites and mosquitoes, but not present in mammals. By searching the literature, ten proteins were found to be involved in the pathogenic infection process of *W. bancrofti*. In addition, the miRNA–gene network analysis indicated that two pathogenic genes (*CALR* and *HMGB2*) are regulated by newly identified miRNAs. These genes were supposed to play key roles in the infection mechanism of *W. bancrofti*. In conclusion, our genome-wide analysis provided new clues for the prevention and treatment of *W*. *bancrofti* infection.

## 1. Introduction

Lymphatic filariasis is a neglected tropical disease (NTD), also known as elephantiasis. This disease is caused by parasitic infection, seriously endangering human health and causing economic burdens. According to the World Health Organization, about 51 million people in 47 countries are affected with this disease [1]. The parasites that cause lymphatic filariasis include *Wuchereria bancrofti*, *Brugia malayi*, and *Brugia timori* [2]. These parasites are transmitted by mosquitoes in different genera, such as *Anopheles*, *Aedes*, and *Culex*. *W. bancrofti* is the main pathogen of lymphatic filariasis, with approximately 90% of incidents in total cases [3]. The species *W. bancrofti* was found in tropical and subtropical regions of Asia, Africa, and the Americas [4]. When mosquitoes carrying infectious larvae bite people, the infectious larvae escape from the lower lip of the mosquito. They then transfer from the wound where the mosquito bites to the human body, and move to the lymph vessels to develop into adult worms. Adult worms dwell in lymph nodes and vessels, producing microfilariae that trigger local and systemic inflammatory responses [5]. This manifests as lymph stasis, lymphedema, and lymphangitis. The disease has significant public health implications due to its potential to cause chronic disabilities and its impact on affected communities, necessitating control measures to prevent and manage the condition [6]. Existing drugs for the treatment of lymphatic filariasis are diethylcarbamazine, ivermectin, and albendazole. While these medications are effective at eliminating microfilariae, the larval stage of the parasite, they have a limited impact on adult worms, which are responsible for the chronic symptoms of lymphatic filariasis [7]. Currently, no effective vaccines are available for the prophylaxis of lymphatic filariasis. While medications like diethylcarbamazine [8] and doxycycline [9] can manage the disease by targeting microfilariae and the bacteria *Wolbachia* within adult worms, respectively, they do not completely eradicate the adult worms. Therefore, the development of more effective drugs and safer treatment options remains a critical need in the fight against this disease.

MicroRNAs (miRNAs) are a class of endogenous non-coding small RNAs approximately 18 to 25 nucleotides long, derived from the stem region of precursor miRNAs (pre-miRNAs) [10]. These miRNAs are involved in the regulation of post-transcriptional gene expression in plants and animals. miRNAs are quite conserved in species evolution and are major regulators of parasite–host interactions [11]. At present, there are few studies on the molecular interaction mechanism of *W. bancrofti* and the host. Given the intricate life cycle of *W. bancrofti*, elucidating the infection mechanisms within its human hosts presents significant challenges. Expressed sequenced tags (ESTs) provide information about the transcriptome [12] and could be used as effective sequences for identifying conserved miRNAs. This EST-based prediction method has frequently been used to identify miRNAs and their targets in a variety of plants and animals, including *Trypanosoma brucei* [13], *Populus trichocarpa* [14], *Mentha piperita* [15], and *Muskmelon* [16]. Despite the significant public threat of *W. bancrofti*, the miRNAs of this pathogen were little described; thus, a systematic investigation of miRNA is in urgent need.

Protein is a biological macromolecule, composed of one or more polypeptide chains, which is the main bearer of life activities. The study of proteins has never stopped, and with the development of whole-genome sequencing (WGS), more and more proteins have been discovered. Scott et al. performed a WGS analysis of *W. bancrofti* larvae and identified some important proteins related to their life cycle [17]. Although WGS technology has accelerated the research process of proteins, there are still many predicted proteins with unclear functions. These proteins are annotated as “uncharacterized proteins”, so re-annotation of these proteins is highly necessary. Homology search is a classical method for predicting protein function, and it is effective and reliable in re-annotating the *W. bancrofti* proteins.

In this study, we used some reliable methods to identify *W. bancrofti* miRNAs and re-annotate uncharacterized proteins. The homolog distribution of proteins in parasites, vectors, and mammals was analyzed. We also explored the regulatory roles of miRNAs and proteins in the infection process, which provided novel clues for the prevention and control of lymphatic filariasis.

## 2. Materials and Methods

### 2.1. Overview of Our Analysis Workflow

We used similar methods reported in previously published studies [13,14,15,16] to identify conserved miRNAs in *W. bancrofti* based on EST sequences. The uncharacterized proteins of the *W. bancrofti* genome were annotated by the homology search method. The flowchart of this study is shown in Figure S1.

### 2.2. Data Collection and Software Information

A total of 4847 *W. bancrofti* EST sequences were retrieved from the NCBI database (https://www.ncbi.nlm.nih.gov/nucleotide/ (accessed on 29 December 2023)). All known animal miRNAs were obtained from the miRBase database (https://www.mirbase.org/ (accessed on 29 December 2023)) [18]. Genome annotation information for the investigated species was downloaded from the UniProt database (https://www.uniprot.org (accessed on 29 December 2023)) [19]. Sequence alignment was conducted by BLAST (version 2.15.0+). BLASTN was used to perform sequence alignment of all animal miRNAs and *W. bancrofti* EST sequences. BLASTX was used to remove protein-coding sequences with an E-value of 1 × 10^−5^. RNAfold is an effective RNA structure prediction tool inside the ViennaRNA web server [20], so we used RNAfold for secondary structure prediction of pre-miRNA with default parameters. In addition, the Jvenn online tool (https://jvenn.toulouse.inrae.fr/app/index.html (accessed on 21 June 2024)) was used to draw Venn diagrams of protein homology distribution [21].

### 2.3. Identify EST Sequences Containing Potential miRNA Sequences

In this study, we utilized a prediction method based on EST homology search to identify novel miRNAs. This approach has been previously validated for its efficacy in discovering miRNAs across various species [13,14,15,16]. The conservation of miRNAs across species evolution facilitated the identification process by allowing us to leverage known miRNA sequences from related species. Our method involves the following steps:

(1) Sequence alignment. To identify potential miRNAs in *W. bancrofti*, we conducted a comparative analysis by aligning miRNA sequences from a diverse range of known animal species against the EST sequences of *W. bancrofti*. This approach enabled us to identify conserved RNA fragments that mapped to the *W. bancrofti* genome, suggesting these regions as candidates for novel miRNAs;
(2) Information extraction. This involved the systematic retrieval of detailed characteristics for each fragment, including the accession number, alignment length, start and end positions within the *W. bancrofti* genome, and the number of mismatches. These data were crucial for further analysis and validation of potential miRNA candidates;
(3) Eliminate redundancy. We conducted a thorough assessment of the alignment results to eliminate redundant data. Specifically, we manually inspected EST sequences that occurred in multiple alignments to ensure that only distinct sequences were preserved. When an individual EST sequence aligned with two or more potential miRNAs, we prioritized the sequences with the fewest mismatches for subsequent analysis.

### 2.4. Secondary Structure Prediction

Because the sequences surrounding the mature miRNAs can be folded to form a stem-loop hairpin structure, the upstream and downstream aligned sequences (miRNA candidates) were extracted and considered as potential pre-miRNA candidates. The secondary structures of these pre-miRNA candidates were predicted by RNAfold to identify stem-loop hairpin structures. Since the length of the precursor sequence is variable, we manually checked the length of these pre-miRNA candidates to find the best hairpin structures.

The following criteria were used to select miRNAs:

(1) The miRNA candidate should not contain N;
(2) The miRNA candidate has less than three mismatches compared to the known miRNA;
(3) The miRNA candidate has the same length compared to the known miRNA, i.e., gap number = 0;
(4) The values of the minimum free energy (MFE) and minimum free energy index (MFEI) of the predicted secondary structure should be significantly negative;
(5) The predicted pre-miRNA is capable of folding into a stem-loop hairpin structure, and the predicted mature miRNA sequence site must be on one arm of the hairpin structure.

These criteria used here are based on previous studies [13,14,15,16]. Screening through these criteria can significantly reduce false-positive results for miRNA identification. By prediction of the secondary structure of these pre-miRNA candidates, MFE values were recorded. The AMFE and MFEI values were also calculated to analyze their significance. AMFE is an adjusted MFE defined as an MFE of 100 nucleotide length. The AMFE and MFEI values for each pre-miRNA were calculated using the following formula:

$$AMFE=\frac{MFE}{Length\;of\;precursor}*100$$

$$MFEI=\frac{AMFE}{(G+C)\%}$$

Since miRNAs are non-coding RNAs, protein-coding sequences need to be removed. The miRNA candidates were aligned with the protein-coding sequences of *W. bancrofti* by BLASTX. To ensure accurate identification of non-coding RNAs, we removed miRNAs with sequences similar to protein-coding sequences. The AMFE and MFEI values are calculated for the remaining sequences. The remaining sequence was considered a pre-miRNA, and the aligned nucleotide sequence was considered a mature miRNA in subsequent analysis.

### 2.5. Homologous Distribution Analysis of W. bancrofti miRNAs

The homologous distribution of miRNAs helps us to understand the conservation and evolutionary relationships among different species, which could reveal the universal role and mechanism of miRNAs in the cell cycle. The *W. bancrofti* miRNAs were searched against all known animal miRNAs to identify their homology. All homology information was integrated, and corresponding figures were plotted.

### 2.6. Analysis of Protein Homology and Functional Annotation

A total of 39,617 *W. bancrofti* proteins were downloaded from the UniProt database, of which 13,926 were annotated as “uncharacterized protein”, accounting for 35.15% of the total proteins. Thus, it was necessary to re-annotate the protein functions of *W. bancrofti*. We performed the protein sequence alignment of *W. bancrofti* and nine other species (*Homo sapiens*, *Mus musculus*, *Rattus norvegicus*, *Aedes aegypti*, *Anopheles gambiae*, *Culex pipiens*, *Culex quinquefasciatus*, *Brugia malayi*, and *Brugia timori*). These species included two parasites causing lymphatic filariasis, four mosquitoes serving as insect vectors, and three mammal hosts. A comparative analysis of the protein homology among these species was also performed. The proteins on these preliminary annotations were then aligned against protein non-redundant sequences (nrs) to improve the annotation accuracy by the BLAST web server at NCBI (https://blast.ncbi.nlm.nih.gov/Blast.cgi (accessed on 18 June 2024)).

### 2.7. Identify Pathogenic Proteins in W. bancrofti

The pathogenic proteins play a crucial role in the occurrence and development of diseases. During the process of *W. bancrofti* infection, many pathogenic proteins are present and play important roles in the parasite–host interactions. The literature related to *W. bancrofti* was searched against the PubMed database (https://pubmed.ncbi.nlm.nih.gov/ (accessed on 1 July 2024)) by the keywords “*Wuchereria bancrofti*” in the Title/Abstract. We then manually checked the literature to collect the reported pathogenic proteins.

### 2.8. Network Analysis

mirTarBase is a database of miRNA target genes focused on experimental validation with strong reliability [22]. TargetScan is one of the commonly used tools for predicting miRNA target genes based on sequence complementarity and thermodynamic stability [23]. miRWalk is a comprehensive miRNA database that provides a wealth of information on miRNA target gene interactions [24]. We used the intersection of the prediction results of these three tools as the final prediction result to improve the accuracy of the prediction. Cytoscape, developed by the Institute for Systems Biology in the USA, is an open-source software platform renowned for its user-friendly and robust network visualization capabilities [25]. In our methodology, we utilized Cytoscape version 3.9.1 to generate interaction networks involving miRNA and genes.

## 3. Results

### 3.1. Overview of miRNA Identification Work

We retrieved 4847 *W. bancrofti* EST sequences from the NCBI database, which were used to construct a library of sequence alignments. All known animal miRNAs obtained from miRBase were used as query sequences for sequence alignment. A total of 37,522 EST fragments were obtained after the BLASTN sequence alignment. After filtering by the criteria of mismatch and gap, 319 EST fragments were left. After removing the redundancy, 193 fragments were left. Among them, 102 starting points were smaller than the endpoint on the alignment, and 91 starting points on the comparison were larger than the endpoint. The upstream and downstream sequence information of these 193 fragments were extracted as miRNA candidates. It should be noted that for sequences with a start point greater than the endpoint on alignment, the reverse complementary sequence needs to be taken as the miRNA candidate. After screening by hairpin structure, a set of 39 fragments remained. These 39 fragments were aligned with the protein-coding sequences of *W. bancrofti* by BLASTX and 22 candidates remained. We will eliminate predicted miRNA sequences that contain ambiguous N nucleotides to ensure data accuracy. Furthermore, sequences with MFE/MFEI values that do not meet the established criteria will be discarded to ensure the accuracy of the identified miRNAs. All of these screening procedures, as well as the number of miRNAs remaining after each criterion, are summarized in Table S1.

In this study, 20 mature miRNAs were finally identified (Table 1). We found that the 20 miRNAs identified were from 20 miRNA families. Each miRNA family has one member. The identified mature miRNAs were 16–23 nucleotides in length, wba-miR-2917 had a minimum length of 16 nucleotides, and wba-miR-6894-5p had a maximum length of 23 nucleotides. The mean miRNA length was 19.65 with a standard deviation of 1.79 (Table S2). The size differences in the identified miRNAs reveal that they may play a role in various functional processes that regulate miRNA biogenesis or gene expression [26]. The number of mismatches can reflect the difference between them and matching homologous miRNAs. The mismatch between two miRNAs (wba-miR-619-5p, wba-miR-1973) is 0, indicating that their miRNA sequences are completely consistent with their matching sequences. The mismatch number of four miRNAs is 1, the mismatch number of ten miRNAs is 2, and the mismatch number of four miRNAs is 3. To show the details of the mismatch, the sequence alignment of five miRNAs and their corresponding homologous sequences were plotted (Figure S2).

### 3.2. Secondary Structure of miRNA in W. bancrofti

To better validate the newly identified miRNAs in *W. bancrofti*, we calculated various parameters such as precursor length, A + U content, G + C content, minimum free energy (MFE), and minimum free energy index (MFEI) for each miRNA (Table 2).

The AU content ranged from 39.73% to 80.00%, with a mean of 52.85%. The GC content ranged from 20.00% to 60.27%, with a mean value of 46.57%. We found that the AU content was higher than the GC content and the hydrogen bonds between the AU pairs were weaker than those between the GC pairs, which may contribute to the pre-miRNAs being more easily cleaved into mature miRNAs during processing and maturation.

**Figure 1:**
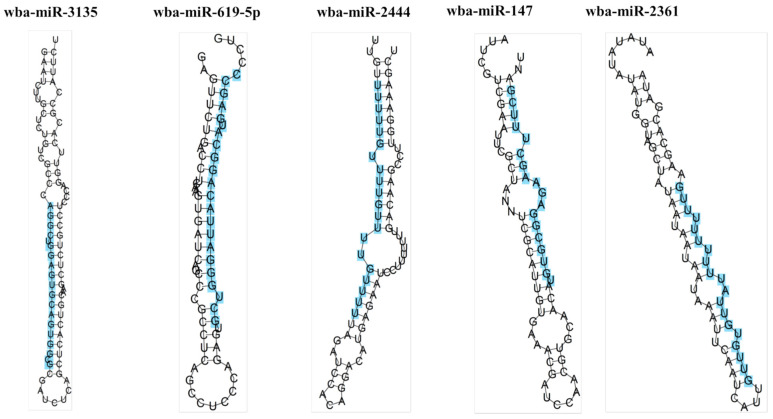
Five identified miRNA hairpin structures of *W. bancrofti*. The sequence highlighted in blue is the mature sequence of the miRNA.

The MFE is an important parameter for determining the secondary structure of pre-miRNAs and is an important feature for miRNA identification. The smaller the MFE, the more stable the miRNA secondary structure [27]. The MFE of the 20 identified pre-miRNAs ranged from −40.40 kcal/mol~−6.50 kcal/mol, with a mean of −20.82 kcal/mol and a standard deviation of 9.20 kcal/mol. Because the value of MFE is length-dependent and will be affected by length, AFME is needed. AMFE is an adjusted MFE defined as 100 nucleotide length. The AMFE range of these 20 pre-miRNAs was −47.34 kcal/mol~−9.29 kcal/mol, with a mean of −25.57 kcal/mol and a standard deviation of 10.16 kcal/mol. Zhang et al. proposed the concept of a minimum free energy index, which provides a standard for the comparison of pre-miRNAs of different lengths [28]. The MFEI range of these 20 pre-miRNAs was (−0.83)~(−0.36), the mean value was −0.54, and the standard deviation was 0.12. The secondary structures of five miRNAs with good stem-loop structures were plotted (Figure 1).

### 3.3. Homologous Distribution of W. bancrofti miRNAs

After identifying 20 miRNAs in *W. bancrofti*, the homologous miRNAs of known animals were retrieved and a distribution table was plotted (Table 3). We found these miRNAs from 17 species, including seven mammals and 10 non-mammals. However, 14 of these homologous miRNAs are from seven mammals, and only six miRNAs are from 10 non-mammals. We found that most of the homologous miRNAs were of mammalian origin, with four homologous miRNAs found in the human genome and four other homologous miRNAs found in the mouse genome. Most miRNAs were found to have homology in only one species, suggesting that most of the *W. bancrofti* miRNAs we have identified are relatively rare among animal miRNAs. Only three miRNAs (miR-1973, miR-153-5p, let-7e-5p) were found in more than one species. Interestingly, although *W. bancrofti* is a non-mammal, it has less homology with non-mammals than mammals. *W. bancrofti* belongs to nematodes with a great difference from most non-mammals with known miRNA, while many nematodes’ miRNA has not been recognized. Notably, a homologous miRNA (miR-10093-5p, miR-755-3p) was found in *Heligmosomoides polygyrus* and *Schistosoma mansoni*, respectively, and none of the homologous miRNAs were found in other species. *Heligmosomoides polygyrus* and *Schistosoma mansoni* are also predominant parasites affecting humans, so we speculate that these two miRNAs may play a key role in the infection of *W. bancrofti*.

### 3.4. Protein Homology Distribution

**Figure 2:**
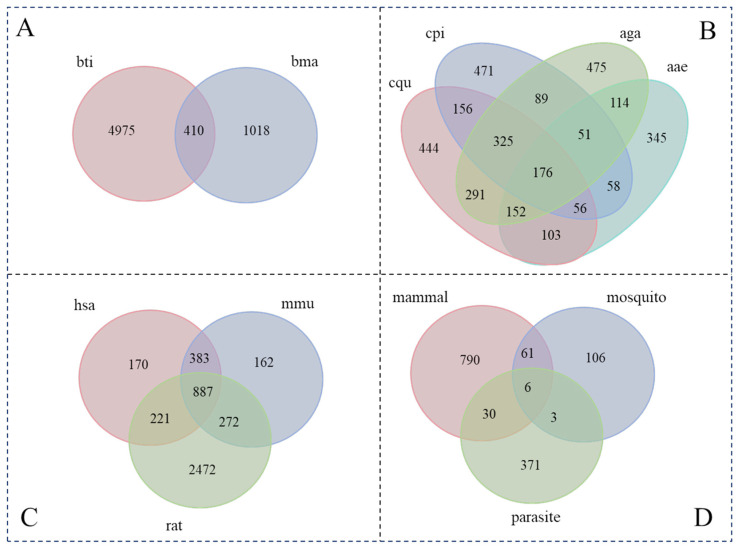
Venn diagrams of *W. bancrofti* homologous proteins in different groups. (**A**) Venn diagram of homologs in two parasites (*Brugia malayi* and *Brugia timori*); (**B**) Venn diagram of homologs in four mosquito vectors; (**C**) Venn diagram of homologs in three mammals; (**D**) Venn diagram of parasite, mosquito, and mammal.

The protein homology of *W. bancrofti* was searched by BLASTP with a bidirectional best-hit strategy against nine species (*Homo sapiens*, *Mus musculus, Rattus norvegicus, Aedes aegypti, Anopheles gambiae, Culex pipiens, Culex quinquefasciatus, Brugia malayi,* and *Brugia timori*). The number of homologous proteins obtained for each species is summarized in Table 4. To better investigate the percentage of homologous proteins in each species, we plotted the percentage of homologous proteins (Figure S3). Interestingly, *Brugia malayi* and *Brugia timori* are both parasites that cause lymphatic filariasis and are transmitted by mosquitoes. However, the percentage of homologous proteins between *Brugia malayi* and *W. bancrofti* is only 3.6%, while the percentage of homology between *Brugia timori* and *W. bancrofti* is 27.62%. In addition, all homologous proteins were analyzed to identify the distribution difference among three groups: parasite, mosquito (vector), and mammal (host), which are plotted with a Venn diagram using Jvenn (Figure 2). We found that three proteins (Ubiquitin-related modifier 1, Mitochondrial uncoupling protein 4, T-complex protein 1 subunit theta) were present in parasites and mosquitoes, but not present in mammals. These three proteins may be used as novel drug targets in the treatment of lymphatic filariasis.

### 3.5. Protein Functional Annotation

We first annotated *W. bancrofti* proteins through preliminary protein sequence alignment by BLAST against the above-mentioned nine species, obtaining 127 potential homology proteins. Then, we searched against a non-redundant protein database by BLAST online tool in NCBI and annotated 75 uncharacterized proteins (Table S3). The homology among these proteins was consistently high, with all identities exceeding 70%, and the majority surpassing 90%. The top 15 annotations are shown in Table 5. The protein A0A1I8EHN2 was currently annotated as “uncharacterized protein” in the UniProt database (updated to July 2024). By our alignment method, we have successfully identified XP_001891657.2 as its protein homolog. The identity of the sequence alignment was 100.00% and the E-value was 2E-140. Thus, protein A0A1I8EHN2 could be annotated as “Ras-related protein Rac1” (RAC1). These results indicate that our results were very robust in the protein annotation process.

### 3.6. Pathogenic Proteins in W. bancrofti

The pathogenic proteins play an important role in parasite–host interactions. We collected 10 disease-related proteins of *W. bancrofti* described in the published papers and their information was shown in Table 6. Detailed information has been included in Table S4. MIF-2 has been reported to be one of the regulatory molecules secreted by *W. bancrofti* and plays an important role in the regulation of the complex host immune system [29]. CST3 is an important immunomodulatory protein that maintains anti-inflammatory immune homeostasis and prolongs parasite survival in the host [30]. GST is a key enzyme responsible for detoxification, and by neutralizing these toxic substances, it is thought to counteract host-mediated oxidative stress responses. SOD is a metabolic enzyme that plays an important role in eliminating superoxide from parasite cells [31]. This inhibition of enzyme activity will lead to the death of the parasite. TCTP can induce eosinophilia and release histamine from mast cells and may play an important role in the pathogenesis of filariasis and allergic inflammation [31]. MFP acts as a ligand for macrophage Toll-like receptor 4 to induce inflammation through the NF-kB signaling pathway [32]. GAL helps to downregulate the pro-inflammatory Th1 response and stimulates the Th2 response, thereby contributing to the long-term survival of the parasite [33]. IPGM is also considered as an important gene for the survival of nematodes [34].

### 3.7. Results of Network Analysis

**Figure 3:**
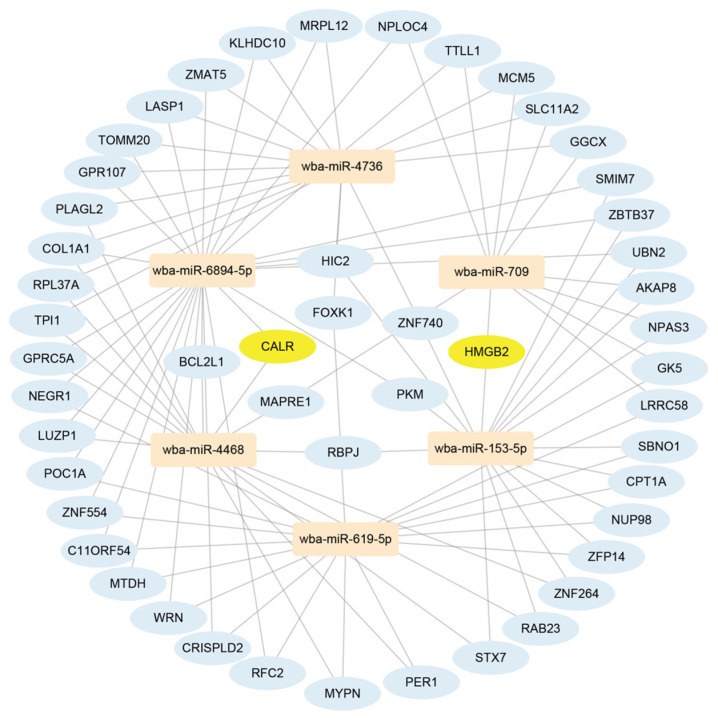
The miRNA–gene interaction network diagram. The miRNAs are shown by light orange nodes, pathogenic genes are shown by light yellow nodes and other genes are shown by light blue nodes.

The targeted genes of these 20 miRNAs were predicted by three databases (mirTarBase, TargetScan, and miRWalk). By the intersection of results in these three databases, nine miRNAs and 925 genes were obtained. A total of 102 genes co-regulated by two or more miRNAs were screened, and these genes were found to be regulated by six miRNAs. We used Cytoscape to construct the miRNA–gene interaction network to study its regulatory mechanisms, as shown in Figure 3.

In this network, we find two key nodes, *CALR* and *HMGB2*. *CALR* has been reported to be a pleiotropic molecule involved in fecundity, infectivity, and regulation of the host immune response. The complement system is an important humoral immune mechanism for defense against pathogens, especially parasites. *CALR* contributes to parasite infection by interfering with the initial phase of host complement activation [35]. *HMGB1* and *HMGB2* are both members of the HMGB family in *W. bancrofti*, and previous studies have shown that the filarial *HMGB1* protein binds to DNA and induces macrophages to secrete pro-inflammatory cytokines [36]. This filarial protein may play an important role in the inflammatory immune response associated with lymphoid pathology. The B-box domain of *W. bancrofti HMGB1* is a potent pro-inflammatory protein. Given its pro-inflammatory function, we believe that HMGB2 may play a role in the pathology of lymphatic filariasis. The network diagram shows that *CALR* is co-regulated by two miRNAs, wba-miR-4468 and wba-miR-6894-5p. *HMGB2* is co-regulated by two miRNAs, wba-miR-153-5p and wba-miR-709. The results showed that four miRNAs, wba-miR-4468, wba-miR-6894-5p, wba-miR-153-5p, and wba-miR-709, played a regulatory role in the infection mechanism of *W. bancrofti* by regulating immune-related genes.

## 4. Discussion

*W. bancrofti* is a parasite that causes lymphatic filariasis, which is transmitted by mosquitoes and poses a danger to human health. Currently, there are very few drugs available to treat lymphatic filariasis, and frequent use is prone to drug resistance [37]. Therefore, there is still a need to find safe and effective treatments for this disease. It is essential to analyze the genome and proteins of *W. bancrofti* to uncover the molecular mechanisms of its infection, which could lead to the development of novel therapeutic strategies and a deeper understanding of the pathogenesis of lymphatic filariasis. The miRNAs are involved in the regulation of post-transcriptional gene expression in animals and plants. Identifying new miRNAs can further understanding of the regulatory role of miRNAs and provide new ideas for the diagnosis and treatment of diseases.

miRNAs are quite conserved in species evolution and play an important role in the regulation of pathogen–host interactions. In previous studies, the EST-based method was widely considered an effective approach for identifying novel miRNAs within eukaryotic genomes [13,14,15,16]. Thus, the screening criteria in these previous studies were also used in this study. A set of 20 miRNAs belonging to 20 different families were identified in *W. bancrofti*. The identified mature miRNA is 16–23 nucleotides in length, which is consistent with the results shown by Bortoluzzi et al. [38] that the mature miRNA length may be between 16–27 nucleotides. The identified pre-miRNAs are 70–108 nucleotides in length. The pre-miRNAs reported by Tili et al. [39] ranged from 70 to 100 nucleotides; Jike et al. [40] reported pre-miRNAs ranging from 60 to 193 nucleotides. The pre-miRNA lengths we identified were similar to previous studies. The ability of pre-miRNAs to fold to form stem-loop hairpins is one of the important conditions for miRNA maturation, and we predicted the secondary structure and demonstrated the stable structure of five miRNAs.

However, the ability to form a stem-loop secondary structure is not unique to miRNAs, and tRNAs, mRNAs, and rRNAs can also have similar hairpin structures. Therefore, other characteristics are needed to further identify miRNAs to avoid misidentifying other RNAs as miRNAs. MFE is a critical parameter for assessing the stability of miRNA secondary structures; a lower MFE value indicates a more thermodynamically stable miRNA structure, which is essential for its recognition and functionality. Zhang et al. [41] reported MFE values ranging from −131.50 kcal/mol~−5.20 kcal/mol, while Curcio et al. [42] reported MFE values ranging from −33.30 kcal/mol~−7.60 kcal/mol. The MFE range of the miRNAs we identified was −40.40 kcal/mol~−6.50 kcal/mol, with a mean value of −20.82 kcal/mol, which is consistent with their research results or within their range. The MFEI serves as a comparative criterion for pre-miRNAs of varying lengths. In this study, the MFEI ranged from (−0.83)~(−0.36), and the mean value was −0.54. In previous miRNA research, Singh et al. [43] reported the MFEI range from (−0.72)~(−0.45), which is a similar range compared to our study. Additionally, the AU content ranged from 39.73% to 80.00%, and the mean value was 52.85%. The GC content ranged from 20.00% to 60.27%, with a mean value of 46.57%. The average AU content of 52.85% was higher than the average GC content of 46.57%. The average value of AU content was higher than that of GC content. The higher the AU content, it is easily recognized by the RNA-induced silencing complex (RISC) and converted to mature miRNA. The GC content of this study ranged from 18% to 86% reported by Gul et al. [44], which supports the results of this study. These features indicated that the *W. bancrofti* miRNA identified in this study is very reliable.

At present, there are still 13,926 *W. bancrofti* proteins with unknown function, accounting for 35.15% of the total number of proteins. By sequence alignment with a non-redundant database, we have successfully re-annotated the functions of 75 uncharacterized proteins. We conducted sequence alignments of the *W. bancrofti* protein against two other parasites (*Brugia malayi* and *Brugia timori*) causing lymphatic filariasis, four mosquitoes (*Aedes aegypti*, *Anopheles gambiae*, *Culex pipiens*, *Culex quinquefasciatus*) serving as insect vectors, and three hosts (*Homo sapiens*, *Mus musculus*, *Rattus norvegicus*). By a bidirectional best-hit strategy, we calculated the percentage of homologous proteins for each species in comparison to *W. bancrofti*. Interestingly, both *Brugia malayi* and *Brugia timori* are parasites that can cause lymphatic filariasis, but the homology percentage of *Brugia timori* and *W. bancrofti* (27.62%) is much higher than that of *Brugia malayi* (3.60%). This result indicated that *W. bancrofti* is more similar to *Brugia timori* in genome evolution.

At present, there are no studies on *W. bancrofti* miRNA and its regulatory role in the infection mechanism. In our investigation of the infection mechanisms of *W. bancrofti*, we have identified 10 proteins that are implicated in the disease process, contributing to our understanding of the parasite’s interaction with the host. In the miRNA-gene network analysis, we found that CALR and HMGB2 were regulated by our identified miRNA. CALR is co-regulated by wba-miR-4468 and wba-miR-6894-5p. HMGB2 is co-regulated by wba-miR-153-5p and wba-miR-709. CALR is a pleiotropic molecule involved in the regulation of the host immune response, interfering with the initial phase of host complement activation and thus contributing to parasite establishment [45]. HMGB proteins are nuclear factors involved in chromatin remodeling and transcription regulation. These proteins contain a B-box domain, which is related to pro-inflammation in *W. bancrofti* infection [46]. These miRNAs play regulatory roles in the mechanism of *W. bancrofti* infection by regulating the pathogenicity proteins CALR and HMGB1. This result not only reveals the regulatory role of *W. bancrofti* miRNA in the occurrence and development of lymphatic filariasis, but also provides potential targets and strategies for the treatment of lymphatic filariasis.

In this study, we discovered that CALR and HMGB2, two proteins associated with pathogenesis, are regulated by the miRNAs we identified. However, the specific regulatory mechanisms require further investigation. In light of the current lack of an effective vaccine to prevent disease caused by *W. bancrofti*, we intend to conduct subsequent research on vaccine development based on the findings of this study.

## 5. Conclusions

In this study, we performed a comprehensive analysis of the *W. bancrofti* genome and proteome. We identified a set of 20 novel miRNAs. The functions of 75 uncharacterized proteins were also annotated and 10 pathogenic proteins in *W. bancrofti* were collected. We found two genes (*CALR* and *HMGB2*) regulated by the miRNAs play important roles in the mechanism of *W. bancrofti* infection. The identification of these molecules offers potential novel targets for the development of therapeutics against lymphatic filariasis, which could lead to innovative strategies for the prevention and control of *W. bancrofti* infection.

## Figures and Tables

**Table 1:** Sequence information of identified *W. bancrofti* miRNAs.

*W. bancrofti* miRNA	miRNA Sequence	Length	EST Accession	Mismatches
wba-miR-619-5p	GCUGGGAUUACAGGCAUGAGCC	22	CK725669.1	0
wba-miR-1973	ACCGUGCAAAGGUAGCAUA	19	CD455789.1	0
wba-miR-2361	GUUGUGUUAUUUUUUUUUG	19	CD374128.1	1
wba-miR-3135	AGGCUGGAGUGCAGUGGCG	19	CK726218.1	1
wba-miR-2917	AUGAAUGACAUGGACU	16	CD374529.1	1
wba-miR-709	GGAGGCUGAGGCAAGAGGA	19	CK850624.1	1
wba-miR-147	UGUGCGGAGAAGCUUUCG	18	CD374323.1	2
wba-miR-4000b-1-3p	UCCUUUGCAACAGGUUUUGC	20	CD374779.1	2
wba-miR-6894-5p	AGGAGGAUGGAGAACUGGGACAG	23	CD374262.1	2
wba-miR-755-3p	UGACAUUCAACUAUUUCAAC	20	CK855350.1	2
wba-miR-153-5p	CCAUUUUGGGGAUUUGCAGCU	21	CD455816.1	2
wba-miR-103-5p	GCCUCCUGACGGUGCUGC	18	CD454997.1	2
wba-miR-4468	AGAGCCGAAGGAUGUGAU	18	CD374125.1	2
wba-miR-4736	AGGCCAGUUAUCUGGGCU	18	CK850118.1	2
wba-miR-6241	CACGGGGGCUGGAAAUCC	18	CK850101.1	2
wba-miR-7010-3p	GGUUCUCCUUUGCUCUGCAG	20	CD455797.1	2
wba-miR-1198-3p	AAGCUGGUCUCUAACUCCUGGC	22	CK850143.1	3
wba-let-7e-5p	UGAGGUAAUAGGUUGAUUAAUU	22	CD455426.1	3
wba-miR-2444	UUUUUGUUUUGUUUUGUUUU	20	CD374705.1	3
wba-miR-10093-5p	UGCGGUUCCGAGAAGCAACUU	21	CK850105.1	3

**Table 2:** Characteristics of 20 precursor miRNAs in *W. bancrofti*. PL: precursor length; (AU)%: (A + U) content percentage; (GC)%: (G + C) content percentage; MFE: minimum free energy (kcal/mol); AMFE: adjust the minimum free energy (kcal/mol); MFEI: Minimum Free Energy Index.

miRNA	Nucleotide Number	PL	(AU)%	(GC)%	MFE	AMFE	MFEI
wba-miR-147	A(18)U(18)G(16)C(15)N(3)	70	51.43	44.29	−18.1	−25.86	−0.58
wba-miR-2361	A(25)U(31)G(9)C(5)N(0)	70	80.00	20.00	−6.5	−9.29	−0.46
wba-miR-4000b-1-3p	A(15)U(33)G(15)C(20)N(0)	83	57.83	42.17	−14.6	−17.59	−0.42
wba-miR-6894-5p	A(20)U(19)G(29)C(24)N(0)	92	42.39	57.61	−34.7	−37.72	−0.65
wba-miR-755-3p	A(32)U(17)G(10)C(11)N(0)	70	70.00	30.00	−10.5	−15.00	−0.50
wba-miR-153-5p	A(15)U(24)G(15)C(15)N(2)	71	54.93	42.25	−17.5	−24.65	−0.58
wba-miR-3135	A(13)U(21)G(22)C(29)N(0)	85	40.00	60.00	−27.8	−32.71	−0.55
wba-miR-103-5p	A(18)U(17)G(25)C(18)N(0)	78	44.87	55.13	−19.2	−24.62	−0.45
wba-miR-1198-3p	A(14)U(18)G(17)C(24)N(0)	73	43.84	56.16	−27.3	−37.40	−0.67
wba-let-7e-5p	A(37)U(36)G(15)C(15)N(0)	103	70.87	29.13	−11.2	−10.87	−0.37
wba-miR-619-5p	A(14)U(15)G(19)C(25)N(0)	73	39.73	60.27	−18.7	−25.62	−0.43
wba-miR-1973	A(21)U(19)G(19)C(15)N(0)	74	54.05	45.95	−21.1	−28.51	−0.62
wba-miR-2917	A(19)U(25)G(13)C(13)N(1)	71	61.97	36.62	−13.8	−19.44	−0.53
wba-miR-4468	A(17)U(24)G(29)C(18)N(0)	88	46.59	53.41	−23.1	−26.25	−0.49
wba-miR-4736	A(30)U(20)G(16)C(22)N(0)	88	56.82	43.18	−21.5	−24.43	−0.57
wba-miR-6241	A(20)U(25)G(38)C(25)N(0)	108	41.67	58.33	−40.4	−37.41	−0.64
wba-miR-7010-3p	A(11)U(37)G(22)C(27)N(3)	100	48.00	49.00	−17.8	−17.80	−0.36
wba-miR-709	A(18)U(16)G(23)C(22)N(0)	79	43.04	56.96	−37.4	−47.34	−0.83
wba-miR-2444	A(15)U(34)G(16)C(10)N(0)	75	65.33	34.67	−10.1	−13.47	−0.39
wba-miR-10093-5p	A(15)U(16)G(25)C(15)N(0)	71	43.66	56.34	−25.1	−35.35	−0.63

**Table 3:** Homology distribution of miRNAs among *W. bancrofti* and other animals. In the table, “P” stands for Present, i.e., the corresponding homologous miRNAs were present in this species. If the shade color of P is yellow, the species is mammal, and if the shade is green, the species is non-mammal. Species abbreviations: hsa, *Homo sapiens*; mmu, *Mus musculus*; rno, *Rattus norvegicus*; bta, *Bos Taurus*; cja, *Callithrix jacchus*; oan, *Ornithorhynchus anatinus*; cgr, *Cricetulus griseus*; cin, *Ciona intestinalis*; sma, *Schistosoma mansoni*; hpo, *Heligmosomoides polygyrus*; cpi, *Chrysemys picta*; gmo, *Gadus morhua*; xla, *Xenopus laevis*; aca, *Anolis carolinensis*; cli, *Columba livia*; pbv, *Python bivittatus*; xtr, *Xenopus tropicalis*.

Homologous miRNA	Mammal							Non-Mammal									
	hsa	mmu	rno	bta	cja	oan	cgr	cin	sma	hpo	cpi	gmo	xla	aca	cli	pbv	xtr
hsa-miR-619-5p	P	-	-	-	-	-	-	-	-	-	-	-	-	-	-	-	-
hsa-miR-1973	P	-	-	-	-	-	P	-	-	-	-	-	-	-	-	-	-
bta-miR-2361	-	-	-	P	-	-	-	-	-	-	-	-	-	-	-	-	-
cja-miR-3135	-	-	-	-	P	-	-	-	-	-	-	-	-	-	-	-	-
bta-miR-2917	-	-	-	P	-	-	-	-	-	-	-	-	-	-	-	-	-
rno-miR-709	-	-	P	-	-	-	-	-	-	-	-	-	-	-	-	-	-
oan-miR-147	-	-	-	-	-	P	-	-	-	-	-	-	-	-	-	-	-
cin-miR-4000b-1-3p	-	-	-	-	-	-	-	P	-	-	-	-	-	-	-	-	-
hsa-miR-6894-5p	-	P	-	-	-	-	-	-	-	-	-	-	-	-	-	-	-
sma-miR-755-3p	-	-	-	-	-	-	-	-	P	-	-	-	-	-	-	-	-
cpi-miR-153-5p	-	-	-	-	-	-	-	-	-	-	P		-	P	P	P	-
gmo-miR-103-5p	-	-	-	-	-	-	-	-	-	-	-	P	-	-	-	-	-
hsa-miR-4468	P	-	-	-	-	-	-	-	-	-	-	-	-	-	-	-	-
hsa-miR-4736	P	-	-	-	-	-	-	-	-	-	-	-	-	-	-	-	-
mmu-miR-6241	-	P	-	-	-	-	-	-	-	-	-	-	-	-	-	-	-
mmu-miR-7010-3p	-	P	-	-	-	-	-	-	-	-	-	-	-	-	-	-	-
mmu-miR-1198-3p	-	P	-	-	-	-	-	-	-	-	-	-	-	-	-	-	-
xla-let-7e-5p	-	-	-	-	-	-	-	-	-	-	-	-	P	-	-	-	P
bta-miR-2444	-	-	-	P	-	-	-	-	-	-	-	-	-	-	-	-	-
hpo-miR-10093-5p	-	-	-	-	-	-	-	-	-	P	-	-	-	-	-	-	-

**Table 4:** The percentage of protein homologs between *W. bancrofti* and other related species. CI: confidence interval.

Group	Association	Species	Homolog	Total	Homologs Percentage	95% CI in Similarity Percentage
Mammal	Infected host	*Homo sapiens*	1661	20,428	8.13%	[46.22%, 47.70%]
		*Mus musculus*	1704	17,184	9.92%	[45.55%, 47.01%]
		*Rattus norvegicus*	3852	20,715	18.60%	[44.66%, 45.58%]
Mosquito	Transmitted vector	*Culex quinquefasciatus*	1703	20,075	8.48%	[46.58%, 48.08%]
		*Culex pipiens*	1382	39,617	3.49%	[45.33%, 46.93%]
		*Anopheles gambiae*	1673	26,179	6.39%	[45.55%, 47.03%]
		*Aedes aegypti*	1055	39,617	2.66%	[43.55%, 45.33%]
Filaria	Cause same disease	*Brugia timori*	5385	19,496	27.62%	[91.54%, 91.98%]
		*Brugia malayi*	1428	39,617	3.60%	[89.87%, 90.83%]

**Table 5:** Top 15 protein annotations in *W. bancrofti*. Currently, all of the proteins in this table are annotated as “uncharacterized protein” in July 2024.

Protein	Homolog	Gene Symbol	Our Annotation	Identity
A0A1I8EHN2	XP_001891657.2	RAC1	Ras-related protein Rac1	100.00%
J9EGK5	MCP9257968.1	RAB2	Ras-related protein Rab-2	99.53%
A0A1I8EH73	KAK6101950.1	SEL1	Sel1 repeat family protein	98.87%
J9EZZ7	KAK6106685.1	HMBOX	Homeobox domain family protein	98.64%
J9FC50	XP_042932147.1	RAB30	Ras-related protein Rab-30, putative	98.38%
J9AVT2	KAK6112434.1	IER	Immediate early response protein (IER) family protein	98.25%
A0A1I8EA78	KAK6111993.1	TMCC2	Putative transmembrane and coiled-coil 2 family protein	97.40%
A0A3P7EC68	KAK6110404.1	ARHGDIB	Rho GDP-dissociation inhibitor 2	96.94%
J9EDE1	XP_042936681.1	MRPS30	28S ribosomal protein S30, mitochondrial, putative	96.20%
A0A3P7EHJ5	KAK6101881.1	RPN13	Proteasome complex subunit Rpn13	96.19%
J9F9J0	KAK6101659.1	RPS18	Ribosomal protein S18 family protein	96.05%
A0A183XMK3	XP_001894312.1	MTX1	Metaxin 1 homolog, putative	95.91%
A0A1I8ERM1	KAK6107604.1	MEMO1	Protein MEMO1	95.50%
J9EIB4	XP_042936799.1	NTF2	Nuclear transport factor 2 (NTF-2), putative	95.30%
A0A183XB90	XP_042938130.1	MRPS2	28S ribosomal protein S2, mitochondrial, putative	95.16%

**Table 6:** Summary of pathogenic proteins in *W. bancrofti*.

No.	Gene Symbol	Protein Accession	Protein Detail	PubMed ID
1	MIF-2	A0A088FMA5	Macrophage migration inhibitory factor-2	38331083
2	CST3	A0A3P7E9H2	Cystatin domain-containing protein	36586276
3	CALR	A0A3P7DSX6	Calreticulin family protein	33667079
4	GST	E3UV59	Glutathione S-transferase	36797509
5	SOD	A1XI87	Superoxide dismutase	21796387
6	TCTP	J9FBX2	Translationally-controlled tumor protein	11985867
7	MFP	J9AIW9	Major microfilarial sheath protein	30477598
8	GAL	A0A183XCC6	Galectin	32035155
9	HMGB1	J9BAQ5	HMG box family protein	22402610
10	IPGM	B4XK96	Independent phosphoglycerate mutase	22908983

## Data Availability

All data can be found in the manuscript and Supplementary Materials.

## Supplementary Materials

The following supporting information can be downloaded at: https://www.mdpi.com/article/10.3390/pathogens13121088/s1, Table S1: Summary of the miRNA screening process and the remaining number after screening; Table S2: Statistics of miRNA precursor characteristic parameters; Table S3: The annotated 75 uncharacterized proteins in this study; Table S4: Full table of identified pathogenic-related proteins; Figure S1: Flowchart of this study; Figure S2: Sequence alignment of identified miRNA; Figure S3: Protein homology in different species.
