# Supplementary figures and images for: Genome-Wide Analysis Reveals Key Genes and MicroRNAs Related to Pathogenic Mechanism in Wuchereria bancrofti
Source: Pathogens. 2024 Dec 10;13(12):1088. doi: 10.3390/pathogens13121088 (PMC11678661; doi:10.3390/pathogens13121088)

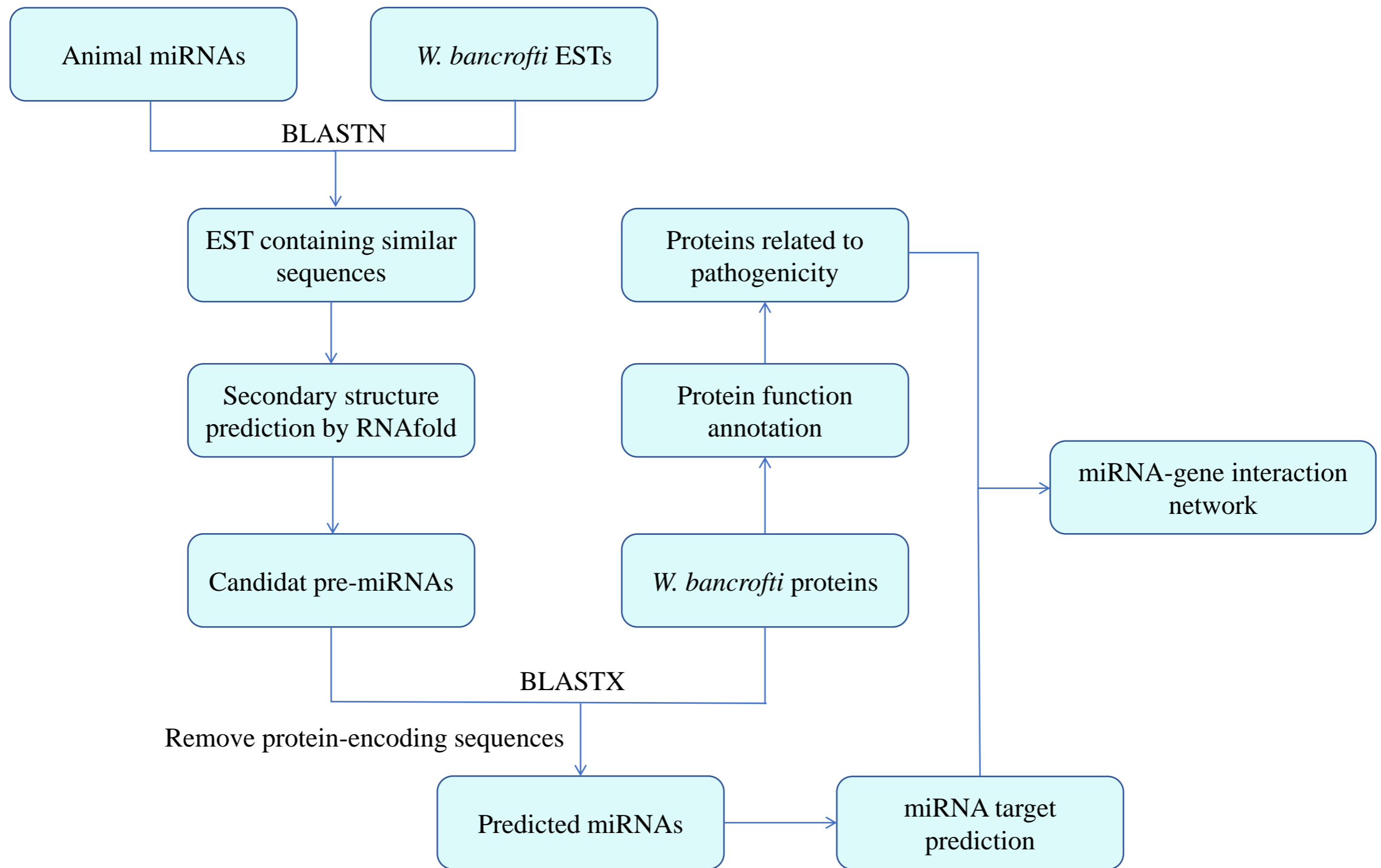

Supplement: Supplementary file 1 [file pathogens-13-01088-s001.zip › pathogens-3318386-supplementary/Supplementary_material_Figure S1 - flowchart of our work.pdf]

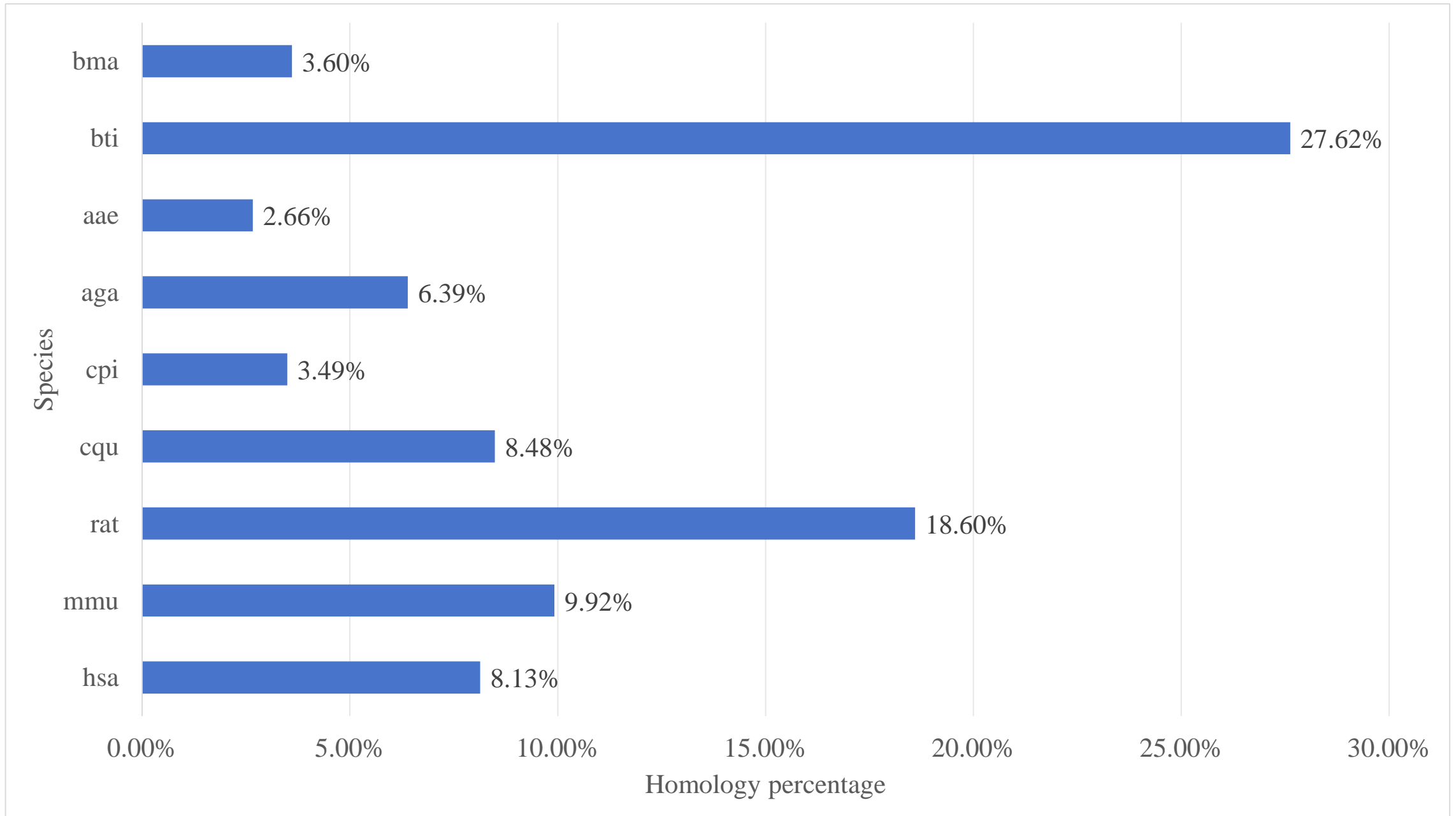

Supplement: Supplementary file 1 [file pathogens-13-01088-s001.zip › pathogens-3318386-supplementary/Supplementary_material_Figure S3 - Protein homology in different species.pdf]
