# Supplementary material for: Genome-Wide Analysis Reveals Key Genes and MicroRNAs Related to Pathogenic Mechanism in Wuchereria bancrofti
Source: Pathogens. 2024 Dec 10;13(12):1088. doi: 10.3390/pathogens13121088 (PMC11678661; doi:10.3390/pathogens13121088)
Supplement: Supplementary file 1 [file Supplementary_material_Figure S2 - sequence alignment of identified miRNA.pdf › pathogens-3318386-supplementary/Supplementary_material_Figure S2 - sequence alignment of identified miRNA.pdf]

A

miRNA		*	*	*	*	*	*	*	*		*	*	*	*	*	*	*	*	*	*
1. wba-miR-3135	-	A	G	G	C	U	G	G	A	G	U	G	C	A	G	U	G	G	C	G
2. cja-miR-3135	A	A	G	G	C	U	G	G	A	C	U	G	C	A	G	U	G	G	C	G

B

miRNA	*	*	*	*	*	*	*	*	*	*	*	*	*	*	*	*	*	*	*	*	*	*
1. wba-miR-619-5p	G	C	U	G	G	G	A	U	U	A	C	A	G	G	C	A	U	G	A	G	C	C
2. hsa-miR-619-5p	G	C	U	G	G	G	A	U	U	A	C	A	G	G	C	A	U	G	A	G	C	C

C

miRNA	*	*	*		*	*	*	*		*		*	*	*	*	*	*	*	*	*
1. wba-miR-2444	U	U	U	U	U	G	U	U	U	U	G	U	U	U	U	G	U	U	U	U
2. bta-miR-2444	U	U	U	G	U	G	U	U	G	U	U	U	U	U	U	G	U	U	U	U

D

miRNA		*	*	*	*	*	*	*	*	*	*		*	*	*	*		*	*
1. wba-miR-147	-	U	G	U	G	C	G	G	A	G	A	A	G	C	U	U	U	C	G
2. oan-miR-147	G	U	G	U	G	C	G	G	A	G	A	U	G	C	U	U	C	C	G

E

miRNA	*	*	*	*	*	*	*	*		*	*	*	*	*	*	*	*	*	*	
1. wba-miR-2361	G	U	U	G	U	G	U	U	A	U	U	U	U	U	U	U	U	U	G	-
2. bta-miR-2361	G	U	U	G	U	G	U	U	G	U	U	U	U	U	U	U	U	U	G	U
